# Books and Authors

**Published:** 1907-08

**Authors:** 


### BOOKS AND AUTHORS.

---

**The Technic of Modern Operations for Hernia.** By **Alexander Hugh Ferguson,** M.B., M.D., Professor of Clinical Surgery, Medical Department of the University of Illinois; Professor of Surgery at the Chicago Post-Graduate Medical School. Royal octavo, pp. 366. Illustrated by sixty-two plates. Chicago: Cleveland Press. 1907. (Cloth $4.00; half morocco $5.00.)

Hernia is, in general, a surgical lesion. It is important that it should be considered by a surgeon of experience. In this treatise the opportunity and the man have met. Ferguson is not only one of our most skilful operating surgeons, but behind the knife he holds so deftly is a keen, discriminate surgical judgment, that makes him competent to deal with difficult questions. When to these is added the ripe experience of twenty years of surgical practice, we may understand how the author is able to prepare such a work as this. He presents the surgical aspect of hernia alone, this affording the only avenue of radical cure at least.

Ferguson divides his book into two parts, the first consisting of twenty chapters. In these he describes technic, instruments and materials used in operations, among which he gives his own technic as employed at the Chicago hospital; he gives the indications for operation, preparation of patient, surgical bacteriology, infection, sterilisation and disinfection, antiseptics and disinfectants, sterilisation of catgut, the wound and its treatment, complications and results, and results from the Ferguson method. He says at the beginning of chapter twenty that "in twenty-four years (1892-1906) I have operated for the cure of hernia in twenty different hospitals," * * * whereas he evidently intended to say fourteen years. Among those who have operated by the Ferguson method he credits John Young Brown, of Saint Louis, with 350 such operations.

In part second the author, in thirteen chapters, describes the various operations for the several forms of hernia. In chapter seven he details his own, which he calls the typic or anatomic operation. Every surgeon should study this chapter as it gives in simple straightforward language a description of the best hernia operation that has been devised up to the present day. The final chapters deal with strangulated hernia, hernia in infants, unusual forms of hernia, and local anesthesia in hernia operations.

Surgical literature pertaining to hernia has been greatly enriched by this book; the illustrations are reproductions of original drawings from the author's collection and make plain the operations which they depict; and the entire volume is a credit to modern American surgery of which the author is one of the greatest exponents.

**A Textbook of Diseases of Women.** By **J. Clarence Webster,** M.D (Edin.) F.R.C.P.E., F.R.S.E., Professor of Obstetrics and Gynecology in Rush Medical College, in affiliation with the University of Chicago. Octavo 712 pages, with 372 text-illustrations and 10 colored plates. Philadelphia and London: W. B. Saunders Company. 1907. (Cloth, $7.00; half morocco, $8.00, net).

The author of this excellent textbook has come to be considered one of the most capable present-day teachers of obstetrics and gynecology. It might be expected that he would produce a good book and it needs but a careful inspection to determine the fact that he has done so. Among the many useful works of this kind, Webster's easily takes its place in the front rank. The treatise is built upon a scientific basis with a solid clinical foundation. The author has a rich clinical field to lay under contribution and he has drawn upon it with discretion. He has gleaned also from modern researches in the laboratory to make his work represent the best as regards diagnosis and treatment. The text is epigrammatic in style without superfluous verbiage, hence is compact in volume, containing the greatest amount of information in the smallest space. The 712 large octavo pages are replete with valuable material which is clearly brought out by the 372 illustrations in the text and the ten colored plates. The artists have been skilful in their work, which compares favorably with the best. It is a treatise that is likely to take a high place as a college textbook and belongs, likewise, on the bookshelves of the advanced practitioner.

**Annual Report of the Surgeon-General of the Public Health and Marine Hospital Service of the United States for the fiscal year 1906.** Washington: Government Printing Office.

No more important public document is issued by the government at Washington than the reports of the public health and marine hospital service, of which the one under present consideration may be regarded as a type. This issue deals with marine hospitals and relief, sanitary reports and statistics, medical inspection of immigrants, foreign and insular quarantine, the fruit port inspection service, domestic quarantine, scientific research and sanitation, besides many other important questions. The inspection of immigrants is made by this service in nearly every port in the world, and is one of the most important functions that it performs. The reports of some of the sanitary officers concerning their inspection of fruit ports furnishes interesting as well as instructive reading. Indeed, the entire book is well worth study by sanitary and public health officers.

---

**Essentials of Obstetrics.** By **Charles Jewett,** M.D., Professor of Obstetrics and Gynecology, in the Long Island College Hospital, Brooklyn, N. Y. Third edition, thoroughly revised. 12mo, 413 pages, with 80 engravings and 5 colored plates. Philadelphia and New York: Lea Brothers & Co. 1907. (Cloth, $2.25, net).

An excellent compend like this one must necessarily be the subject of reissue at reasonably short periods of time. The author is thus enabled to keep his work fresh, adding from time to time whatever is new if important, rejecting what may have become obsolete. Dr. Jewett is recognised as one of our most accomplished teachers, and this book of "Essentials" is accepted as one of the best of its class. Such books, in the present conditions of medical teaching, have become a necessity, hence there is no apology needed from an author who presents a scientific condensation of obstetrics, as Jewett has done. The work is likely to become a classic.

**The Practical Medicine Series of Year Books.** Ten volumes. Issued under the general editorial charge of **Gustavus P. Head,** M.D., Professor of Laryngology and Rhinology in the Chicago Post-Graduate Medical School. Vol X. Skin and Venereal Diseases, Nervous and Mental Diseases. Edited by **W. L. Baum, Hugh T. Patrick, William Healy.** Series 1906. Chicago: The Year Book Publishers. (Price, $1.25; entire series, $10.00).

This volume covers the year's work in skin and venereal diseases and nervous and mental diseases, W. L. Baum editing the former and Hugh T. Patrick and William Healy, the latter section. The work is up to the level of former volumes of the series, and is most complete. Nothing of any value has been omitted in any of the departments. The illustrations are excellent and the descriptive matter is intelligently concise.

---

**Transactions of the third and fourth annual conferences of State and territorial Health Officers with the United States Public Health and Marine Hospital Service,** held at Washington, D. C., May 15, 1905, and May 23, 1906. Two volumes. Washington: Government Printing Office.

These two brochures bound in cloth contain excellent material for sanitarians and public health officers to study and ponder. Views were exchanged between the representatives of twenty-nine states in the one instance and twenty-two in the other on various subjects relating to public health, and the conferences reflect the highest credit on Surgeon-General Wyman who planned the meetings, as well as upon those who participated. It is important that all the states and territories shall be represented in future conferences.

---

**The Hygiene of Pregnancy.** By **Walter B. Jennings,** M.D., Attending Physician to St. Mary's Free Hospital for Children. New York: Medical Review of Reviews. (Price, 25 cents).

A primer of this kind is capable of accomplishing much good if judiciously distributed to the laity. It is particularly designed for a woman about to become a mother, and can be read by such a person with great benefit. A physician, through this medium, may afford his patients much valuable instruction with economy of time. Jennings has written plainly and wisely. His work is deserving of high commendation.

